# Medullary Sarcoma in an Infant

**Published:** 1849-02

**Authors:** 


*Medullary Sarcoma in an Infant.*—M. Blaine narrates the case of an infant, aged nine months, who became the subject of this formidable disease. When two months old, a lump, the size of a pea was observed at the inner part of the right thigh ; six weeks after a second tumour appeared in the corresponding groin, and the two coalesced ; two months after the disease also showed itself in the right shoulder. At the present time the tumour extends completely across the pelvis, involving the right thigh and buttock, the scrotum and penis. It is tense, elastic, and lobulated ; numerous blood vessels ramify upon its surface. The orifice of the urethra is marked by a slight puckering of the integuments, and the urine is voided with pain and difficulty.—*Med. Times.*

[The fearful extent and appearance of the disease is well represented in a wood-cut. The infant was alive at the time of the report, but was threatened with speedy dissolution.]

